# Health Status and COVID-19 Epidemiology in an Inland Region of Portugal: A Retrospective Study

**DOI:** 10.3390/ijerph21081033

**Published:** 2024-08-06

**Authors:** Jorge Lindo, Patrícia Coelho, Catarina Gavinhos, Manuel Martins, Joana Liberal, António Jorge Ferreira, Teresa Gonçalves, Francisco Rodrigues

**Affiliations:** 1 FMUC—Faculty of Medicine, University of Coimbra, 3004-504 Coimbra, Portugal; lindojorgester@gmail.com (J.L.);; 2 CNC-UC—Centre for Neuroscience and Cell Biology, University of Coimbra, 3004-504 Coimbra, Portugal; 3 CIBB—Centre for Innovative Biomedicine and Biotechnology, University of Coimbra, 3004-504 Coimbra, Portugal; 4 Dermatology Department, Coimbra’s Hospital and University Center, Unidade Local de Saúde de Coimbra, 3004-561 Coimbra, Portugal; 5 Sport Physical Activity and Health Research & Innovation Center (Sprint), Polytechnic Institute of Castelo Branco, 6000-084 Castelo Branco, Portugal; patricacoelho@ipcb.pt; 6 IPCB/ESALD—Instituto Politécnico de Castelo Branco, Escola Superior Agrária, UID-QRural, 6000-084 Castelo Branco, Portugal; gavinhos@ipcb.pt (C.G.); mmartins@ipcb.pt (M.M.); 7 IPC/ESTSC—Instituto Politécnico de Coimbra, Escola Superior Tecnologia da Saúde de Coimbra, 3046-854 Coimbra, Portugal; joanaliberal@gmail.com

**Keywords:** COVID-19, Portugal, Beira Baixa, epidemiology, health

## Abstract

Multiple factors, from socioeconomic development to genetic background, can affect the regional impact of some diseases, and this has also been seen during the COVID-19 pandemic. The objective of this retrospective study was to characterize a population in the interior of Portugal regarding health status and COVID-19 epidemiology. Between October 2021 and January 2023, 1553 subjects residing in Beira Baixa, Portugal, were included. Using a self-report approach, demographic and clinical data were obtained. Blood group, blood pressure, peripheral oxygen saturation and anti-spike protein immunoglobulin concentration were also analyzed. Statistical analysis was performed using IBM SPSS Statistics. The average age of the participants was 48.95 (±14.43) years, with 64% being male and 36% being female. The most prevalent comorbidities were hypertension (19.2%), dyslipidemia (12.6%) and diabetes mellitus (6.6%). Half of the population was overweight, and more than half of the subjects had no history of tobacco consumption. Among the participants, 33% were infected with SARS-CoV-2: 70.1% had mild disease, 14.1% moderate disease and 1.4% severe disease. There was a very significant adherence to vaccination (97%). Previously infected or vaccinated people had higher anti-spike protein immunoglobulin values; this value depended on the vaccine administered (*p* < 0.001). Patients with autoimmune diseases and smokers had lower levels of anti-S IgG antibodies (*p* = 0.030 and *p* = 0.024, respectively). The severity of COVID-19 did not affect the concentration of anti-S IgG (*p* = 0.430). This study highlights the general health statuses and the impact of COVID-19 on a population in the Portuguese interior. Knowledge of the circulation and impact of the virus in this specific population can alert and assist in better interventions being conducted by health authorities.

## 1. Introduction

Populations throughout the world have become progressively healthier. However, this improvement is heterogeneous and not equal, depending on the region. In fact, socioeconomic development, risk factors, interventions, and genetic background, among other factors, may interfere with the incidence, prevalence, and severity of diseases, some of which are associated with the elderly [1].

In fact, the aging of the population, particularly in the Western world, has become a challenge, increasing the burden of chronic diseases. The distribution of these pathologies is variable, due to modifiable factors (such as lifestyle) and non-modifiable factors (such as genetic polymorphisms and mutations). Understanding the epidemiology of some regions, in terms of health, may help identify potential interventions to address multimorbidity [2]. According to a systemic analysis in 2016, in Portugal, life expectancy at birth has improved from 77.88 in females and 70.74 in males in 1990 to 80.96 in females and 78.05 in males in 2016 [3]. In the inland regions of Portugal, less densely populated areas, such as Beira Baixa, the population is aged. Additionally, Guarda and Castelo Branco are included in the group of districts with the least purchasing power, which may be a disadvantage in terms of health outcomes. The distance from geographical areas with better access to healthcare is also a major concern for these populations, who are isolated not only in terms of the number of people/inhabitants but also in terms of access to healthcare, particularly human resources and technical conditions. These data have already been proposed as a possible explanation for variations in the distribution and severity of certain diseases such as in asthma outcomes, since these districts were found to have the highest number of prevalence-adjusted asthma hospital admissions [4]. The aging of the population in the interior of Portugal has also been referred to as a potential susceptibility factor to some conditions, like being overweight and/or having obesity [5]. Additionally, Guarda and Castelo Branco belong to the group of districts in Portugal that have fewer registered physicians, according to data concerning the year of 2022 from the Portuguese Medical Association [6].

In 2019, a pandemic emerged due to the spread of SARS-CoV-2 (Severe Acute Respiratory Coronavirus 2), the virus that causes COVID-19. Clinically, the patients with the virus experience fever, cough, dyspnea, myalgia, pharyngitis, headache, and gastrointestinal symptoms, among others. Regarding analytical results, some parameters were found to be abnormal, with elevated C-reactive protein levels, lymphocytopenia, elevated lactate dehydrogenase and the elevation of AST and ALT. As for radiological findings, multilobular ground-glass opacities have been frequently described. The lethality rate varies from 0.3% to 14% [7]. Some patients do not recover completely, developing post-COVID-19 syndrome or post-COVID-19 conditions, the so-called Long COVID, as defined by the WHO [8].

Symptoms, severity and sequelae of COVID-19 vary significantly in the population. The impacts of intrinsic factors (such as sex, age, genetic background and comorbidities) or extrinsic factors (like environmental exposures) are still not fully understood [9,10,11]. This work aims to characterize a population in the inland of Portugal as regards its health statuses and the COVID-19 pandemic.

## 2. Materials and Methods

### 2.1. Ethical Considerations

The Ethics Committee of the Instituto Politécnico de Castelo Branco approved the execution of this study (33-CE-IPCB2021). All subjects signed a consent form in which they agreed to respond to an inquiry, including providing personal and medical data, according to the instructions of the Ethics Committee, and to allow the collection of a venous blood sample.

### 2.2. Study Design and Subjects

This was a retrospective study that covered 1553 subjects living in Beira Baixa, (Portugal, that were enrolled in this research between October of 2021 and January of 2023. The inclusion criteria comprised being older than 18 years of age, accepting to respond to an inquiry and to have a blood sample collected. Not living in the referred region, being under 18 years of age or being pregnant were the exclusion criteria.

Demographic and clinical data such as sex, age, height, weight, comorbidities and smoking habits were gathered using a self-reporting approach, a methodology previously followed in several studies [12,13]. The same approach was applied regarding the information of previous SARS-CoV-2 infection, including the severity, according to predefined criteria as asymptomatic, mild, moderate, or severe [10]. These criteria were based on the presence/absence of manifestations in general, the presence/absence of dyspnea, the search for medical care and the need to be admitted to a hospital. Information regarding experienced symptoms and vaccination statuses, including the type of vaccine received, was also collected [12,13].

### 2.3. Laboratory Techniques

Venous blood was collected from a peripheral vein into an EDTA-treated tube. Then, a biobank was created with those samples. The blood group was determined using Diagast sera and anti-spike protein immunoglobulin was obtained using the commercial AntiSARS-CoV-2 Elisa kit (Euroimmun, Lüebeck, Germany). Peripheral oxygen saturation was measured with an Oxy-3 pulse oximeter (Ref: GI022.35090024031925 GIMA).

### 2.4. Statistical Analysis

The collected data were used to build a database in Microsoft Excel^®^ for Microsoft 365 MSO (version 2302 Build 16.0. 16130. 20186) 64-bit. Then, IBM SPSS Statistics for Windows version 27.0 (SPSS Inc., Chicago, IL, USA) was used to perform the descriptive and the inferential statistical analyses. The referred programs were also used to design graphics.

For continuous variables, data are presented as mean and standard deviation or a 95% Confidence Interval. For categorical variables, data are presented as absolute and relative frequencies.

To evaluate normality, the Shapiro–Wilk test was used. Regarding inferential analysis, a *p*-value lower than 0.05 was considered to be statistically significant. The Chi-square test and, when indicated, the Fisher Exact test (followed by binary logistic regression) were performed to compare qualitative variables. Kruskal–Wallis and Mann–Whitney tests were performed to compare qualitative versus quantitative variables. Kendall rank correlation was used to compare quantitative variables. To deal with missing data, an available-case analysis (pairwise deletion) was considered.

## 3. Results

### 3.1. Characterization of the Sample

This study included 1553 subjects with a mean age of 48.95 (±14.43) years, comprising 994 males (64%) and 558 females (36%).

Most subjects had a body mass index (BMI) categorized as normal (41.3%) or overweight (37.4%), with a mean BMI of 26.28 kg/m^2^. Only 3% of the subjects were either underweight (1.5%) or severely obese (1.5%). Regarding arterial blood pressure (BP), the mean was 126.01 mmHg and 80.23 mmHg for systolic and diastolic BP, respectively. The mean of peripheral oxygen saturation was 97.75%. Most patients never smoked (56.6%), while 24.5% were former smokers and 18.9% were current smokers. The mean number of cigarettes smoked across the entire population was 14 pack years.

A total of 631 subjects were characterized according to the ABO and Rh blood group systems. The most frequent groups were A+ (41.5%) and O+ (31.7%), while the rarest was AB− (0.5%).

At the time of the interview, 505 (33%) had a previous documented SARS-CoV-2 infection, while 1046 (67%) were never infected by the virus.

The previous results are summarized in Table 1.

### 3.2. Disease Prevalence in the Study Population

As expected, the subjects included in this study presented several pathologies (Table 2). The most prevalent comorbidities were arterial hypertension (19.2%), dyslipidemia (12.6%) and diabetes mellitus (6.6%).

Among patients with arterial hypertension, 88.6% were under medical surveillance and pharmacologically treated, while for the ones with dyslipidemia, only 54.1% were treated (Table 3). Thus, the number of individuals treated for hypertension was not significantly different from those treated for dyslipidemia, although the result was marginal (*p* = 0.052).

Most of the diseases were more common in older people, such as ischemic disorders (in general) (*p* < 0.001), coronary artery disease (*p* < 0.001), cerebrovascular disease (*p* = 0.005), heart failure (*p* < 0.001), arterial hypertension (*p* < 0.001), chronic kidney disease (*p* = 0.003), diabetes mellitus (*p* < 0.001), dyslipidemia (*p* < 0.001), oncologic disorders (*p* = 0.006), thyroid disorders (*p* = 0.029) and obstructive sleep apnea disorder (*p* = 0.007). Contrarily, allergies (*p* = 0.004) and asthma (*p* = 0.017) were found to be more frequent in younger patients. Autoimmune, pulmonary, psychiatric and hematologic disorders in general, as well as arrhythmias, were not found to be associated with age (*p* > 0.05).

The results showed that the women had higher rates of coronary artery disease (*p* < 0.001), diabetes mellitus (*p* = 0.001), and arterial hypertension (*p* = 0.008) compared to the men. Conversely, thyroid disorders were more frequent in the men (*p* < 0.001).

Older individuals presented with being more frequently overweight versus being a normal weight, in comparison with younger people (adjusted significance: *p* < 0.001). Also, systolic and diastolic arterial pressure increased with weight (*p* < 0.001). A higher body mass index (BMI) was associated with individuals with diabetes mellitus (*p* < 0.001), arterial hypertension (*p* < 0.001), dyslipidemia (*p* < 0.001), obstructive sleep apnea (*p* = 0.038), ischemic disorders in general (*p* = 0.050) and cerebrovascular disease (*p* = 0.007). Opposingly, a lower BMI was associated with asthma (*p* = 0.022).

Regarding blood groups, there was an association between AB0Rh with obstructive sleep apnea (*p* = 0.046), with AB+ potentially being a susceptibility factor for this disease (*p* = 0.002; OR = 13.51). Pulmonary diseases, in general, were found to be associated with the B group (*p* = 0.021; OR = 3.02).

### 3.3. COVID-19 Patients and Immunization

As reported before, 505 (33%) individuals had been previously infected with SARS-CoV-2 at the time of the interview. The mean age of these participants was 47.43 (±13.30) years, comprising 309 males (61%) and 196 females (39%). Regarding sex and comorbidities, the infected population was not different from the non-infected population (*p* > 0.05). Nevertheless, non-infected people (mean rank = 800.20) were generally older than infected people (mean rank = 725.88) (*U* = 248.803.5; *p* = 0.002).

Of these, 73 (14.5%) were asymptomatic, 354 (70.1%) presented mild disease, 71 (14.1%) moderate disease and 7 (1.4%) severe disease. Nine patients were admitted to the hospital, two of which were admitted to the Intensive care unit (ICU). These results are summarized in Table 4.

**Figure 1:**
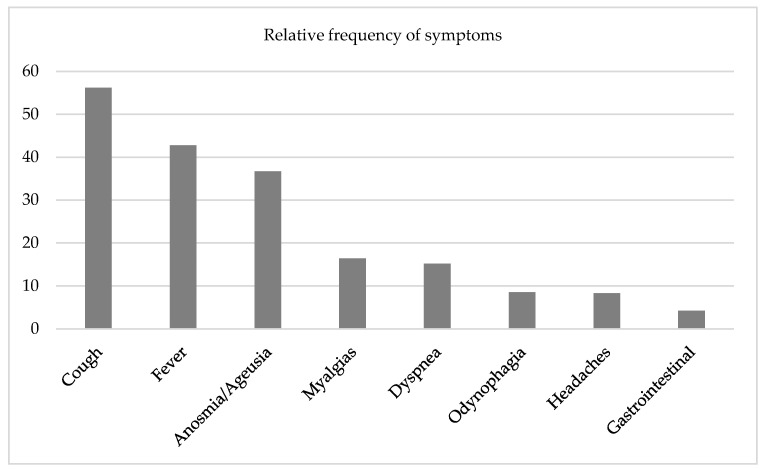
Relative frequency of symptoms during COVID-19 infection.

Among the ones who were symptomatic, the most frequent symptoms were cough (56.2%), fever (42.8%) and anosmia/ageusia (36.7%). Other symptoms were referred to, such as dyspnea, myalgias, gastrointestinal symptoms (like nausea, vomiting and diarrhea), headaches and odynophagia. Figure 1 presents the results concerning symptoms.

Sixteen individuals reported being infected twice: five (31.3%) were asymptomatic, nine (56.3%) had mild disease, two (12.5%) had moderate disease, and none experienced severe disease. Comparing the severity of the second infection with the first one (Table 5), there were statistically significant differences (*p* = 0.038), with lower severity in the second infection. The most common symptoms were cough (37.5%) and fever (31.3%). One person reported being infected three times, with mild disease the third time, experiencing solely anosmia/ageusia and cough. In his two previous infections, he was asymptomatic during the first infection and had mild disease (with fever and myalgias) during the second.

Ninety-seven percent of the individuals included in this study were vaccinated against SARS-CoV-2 infection at the time of the interview. The most common scheme was the administration only of BTN162b2—Pfizer/BioNTech (64.3%), as represented in Table 6. Regarding the technology of the administrated vaccines, 76.5% took gene vaccines (mRNA-1273—Moderna and/or BNT162b2—Pfizer/BioNTech), 9.4% took viral vector vaccines (Ad26.COV2.S—Janssen (Johnson & Johnson) or AZD1222—Oxford/AstraZeneca), 0.1% took whole-virus vaccines (CoronaVac—Sinovac) and 14.10% took combinations of different types of vaccines.

Comparing vaccination status (vaccinated/not vaccinated) with the severity of COVID-19, a non-significant result was obtained (*p* = 0.240—Fisher-Freeman-Halton Exact test); when considering vaccine type (gene, viral vector, mix, whole-virus), a non-significant result was also observed (*p* = 0.713—Fisher- Freeman-Halton Exact test), as was the result for the comparison with the full vaccination scheme according to health authorities’ guidelines (*p* = 0.479—Fisher-Freeman-Halton Exact test).

Blood samples of the participants were tested to evaluate immunity against COVID-19, independently of having been infected or vaccinated previously. Around ninety-two percent (91.9%) were considered immune. The mean concentration of anti-spike (anti-S) immunoglobulin G (IgG) was 366.6 [356.6–376.4] AU/mL. Furthermore, the anti-S IgG concentration was concluded to be different depending on whether a person had been infected/vaccinated or not. In fact, and as expected, people previously infected or vaccinated presented higher values (*p* < 0.001).

As depicted in Table 6, the administered vaccine also caused distinct values of anti-S IgG (*p* < 0.001). Administration of only the Pfizer (*p* < 0.001) or the Moderna (*p* < 0.001) vaccine lead to the development of higher concentrations than the AstraZeneca vaccine. Additionally, the AstraZeneca vaccine alone presented lower values when compared with the Pfizer/AstraZeneca, Moderna/AstraZeneca, Pfizer/Janssen, Moderna/Janssen or Pfizer/Moderna (*p* < 0.001 for all these comparisons) vaccines.

The Janssen vaccine alone also presented consistently lower values, when compared with the Pfizer (*p* < 0.001), Moderna (*p* < 0.001), AstraZeneca/Pfizer (*p* < 0.001), AstraZeneca/Moderna (*p* < 0.001), Pfizer/Moderna (*p* < 0.018), Pfizer/Janssen (*p* < 0.001) or Janssen/Moderna (*p* < 0.001) vaccines.

There were also differences when comparing the Pfizer vaccine with Pfizer/AstraZeneca (*p* = 0.006) or Moderna/AstraZeneca (*p* = 0.004). The referred combinations presented higher concentrations than the Pfizer vaccine alone.

**Figure 2:**
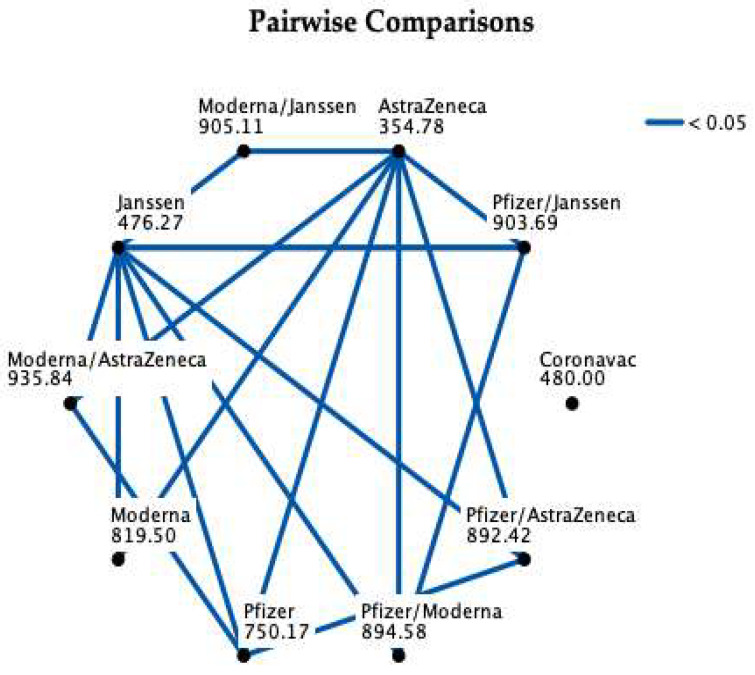
Vaccination data: summary of pairwise comparisons (each node shows the sample average rank of the vaccine).

All the other comparisons of vaccines vs. anti-S IgG concentrations were non-significant. Figure 2 presents a summary of all the pairwise comparisons.

Considering the technology of the vaccine, viral vector vaccines in general lead to the development of lower concentrations of anti-S IgG, in comparison with gene vaccines or combinations of vaccines (*p* < 0.001 for all comparisons). Also, gene vaccines produced lower concentrations of this antibody when compared to combinations of vaccines based on different technologies. These results are presented in Table 7.

In addition, patients with an autoimmune disease presented lower levels of anti-S IgG (*p* = 0.030) and smokers presented lower values than never-smokers (*p* = 0.024). The severity of COVID-19 did not affect the concentration of anti-S IgG (*p* = 0.430).

## 4. Discussion

Worldwide, multiple studies have provided data about health across the globe, as well as about COVID-19 [1,14,15].

This study results from a project developed during several phases of the COVID-19 pandemic with the aim of studying the health statuses of people living in an inland Portuguese area and the epidemiology of SARS-CoV2 infection, together with the response and impact of the vaccination programs.

The mean age of the population under study, inhabiting the region of Beira Baixa, Portugal, was 48.95 years. Considering that the majority of the population in this territory has 15–64 years of age, as observed in the 2021 census, the observed average age is representative of that, although it also presents a high range with people at a very advanced age, characteristic in older rural areas. Additionally, in our study the number of male participants was higher than the number of female participants, which is not concordant with the 2021 census. In fact, in 2021, 47% of people were men and 53% were women in the studied geographic area [16]. It is possible that men have a higher adherence to this type of initiative within the scope of COVID-19, as shown by some studies that analyze long-term COVID, in which most participants were male [17,18].

Our results regarding the BMI showed that more than 50% of the population is overweight, as described in other countries, which proves that health initiatives regarding this matter should be made [19]. In a study carried out in the Portuguese population in 2018, which covered more than 32 thousand people, it was found that 46% were of normal or low weight and around 37% were overweight. This way, the values found in this study reflect a worsening of the overweight status of the Portuguese population, demonstrating that work in this area must be continued [20].

Older individuals had excess weight more frequently in comparison with younger people. It is known that increasing weight is associated with multiple pathologies, namely arterial hypertension, as we concluded in the present study [21]. On the other hand, a lower BMI was associated with asthma. In a study carried out in Canada, it was concluded that a higher BMI and obesity are related to worse asthma control and quality of life, but not to asthma severity [22]. Asthma is a disease with significant prevalence in younger individuals that, in our study, presents a lower BMI which may limit conclusions in this matter.

Analyzing the observed arterial blood pressure (BP) results, it can be considered that this population had average blood pressure levels within the recommended range. These data become even more important because there are reports that chronic pathologies, including high blood pressure, are associated with more serious cases in the context of COVID-19 [23]. It should be noted that 19.2% of the population has hypertension, with more than 88% of these people taking medication.

More than half of the individuals never smoked (56.6%), 24.5% were former smokers and 18.9% were current smokers. Tobacco consumption in Portugal has been decreasing among males (from 35% in 1987 to 26.7% in 2014) and increasing among females (from 6% in 1987 to 14.6% in 2014) [24], but the most recent data from Portugal, obtained in the SICAD study (2017–2022) [25], shows an average percentage of smokers of 51%. Here, we report lower values of smokers, even if we add the people who report being smokers (18.9%) with those who report being ex-smokers (24.5%), since no temporal limit was defined in this case (V National Survey on the Consumption of Psychoactive Substances in the General Population 2022). During the pandemic period, according to a study carried out in Portugal and Spain, there were no changes in tobacco consumption habits [26], so we can consider the values obtained as indicating that the population under study has a lower tobacco consumption than the average number in Portugal [26].

The individuals included in the study presented pathologies of different medical areas. Pathologies were assessed by self-report, as it is a technique used in several other studies about health sciences [10] and COVID-19 [27]. This type of approach allows us to understand the pathologies that participants report, based on their experiences and knowledge. This methodology was also validated in articles that demonstrate that the best way to evaluate COVID-19 in the long term was throuhg self-report methodologies [26]. Arterial hypertension, dyslipidemia and diabetes mellitus were the most frequent comorbidities. The average arterial pressure value found was clearly lower than that found in this region, according to the study of the “ blood pressure program—PPABB”, which has a massive scope in this geographical area, with 40 to 50% of the population presenting arterial hypertension [28,29]. The main explanation for this bias is probably related to the fact that in this work, the participants indicated that they suffered from hypertension (i.e., they had prior knowledge of this fact), whereas in the PPABB program, the researchers carried out the evaluation. 

The estimated prevalence of diabetes in Portugal is around 13.5% [30], clearly higher than the percentage found in our study, as well as dyslipidemia which, according to an extended review study from 2003, shows a prevalence of dyslipidemia of 63.8% (considering a cut-off point of 190 mg/mL of cholesterol) [31]. This indicates that the self-report methodology is a possible technique if the inquired patients have a previous clinical diagnosis and are aware of their health condition. Otherwise, particularly in diseases that may not present immediate symptoms, the values obtained are always considerably lower than those obtained by studies that are based on data collected from clinical files.

The number of individuals with hypertension treated for this disease was not different from the ones with dyslipidemia treated accordingly; however, the result was marginal (*p* = 0.052). These data must be analyzed with concern, since there are individuals who know they have the pathology (dyslipidemia or hypertension), but who choose not to be treated, which may reveal, at the same time, imprudence and a deficit in health literacy. In fact, a study carried out in this context, in 2022, showed that most of the Portuguese health literacy was classified as sufficient (65%), problematic (22%) or inadequate (8%), and only 5% as excellent [32]. Elderly people present more diseases, as expected, since many of these disorders are commonly associated with aging [33,34].

In the population under study, 505 (33%) individuals had been previously infected with SARS-CoV-2, when they were interviewed. Of these, 73 (14.5%) were asymptomatic, 354 (70.1%) presented mild disease, 71 (14.1%) presented moderate disease and 7 (1.4%) presented severe disease. The values obtained are low when compared to several other studies [35,36]. This study lasted a very long time and went through various phases of the pandemic, with the final result being the average of this process. The most frequent symptoms were cough, fever and anosmia/ageusia, among others. These symptoms are always the most frequent mentioned in the various scientific studies reviewed and are usually associated with respiratory infections [37,38].

In our analysis, some individuals were infected twice and there were differences in the severity when comparing the first and the second infection, with reinfection not leading to severe cases. This contrasts with what was found in another study, which reported that during reinfection, patients did not experience fewer symptoms, with the exception of diarrhea, which was more prevalent in the first infection [39].

Ninety-seven percent of the individuals included in this study were vaccinated against SARS-CoV-2 infection. The most common scheme was the administration only of BTN162b2—Pfizer/BioNTech. The most used types of vaccines, as well as the administration schedules, underwent evolutions throughout the initial period; however, a stabilization was then noted, with schemes with messenger RNA vaccines always being the most prevalent [40]. The main highlight is the enormous adherence to vaccination, as demonstrated by more than 97% of the individuals being vaccinated, thus showing that the Portuguese population realized the importance of vaccination and had the means at their disposal to obtain this vaccination [41]. We also show that, in this region, the vaccination status and the vaccine type do not appear to be unique factors explaining the severity of COVID-19, indicating that the severity of this disease is multifactorial.

Blood samples of the participants were tested to evaluate immunity against COVID-19. About ninety-two percent (91.9%) were considered immune and the anti-S IgG concentration was higher in people infected/vaccinated. Immunity against SARS-COV-2 infection is quite high in the world population at the moment; however, it has been evolving in parallel with the pandemic. Naturally, the large increase occurred in line with the vaccination process, since there were two complementary ways of obtaining it—vaccination and natural disease [42]. In our study, the values obtained reveal almost all people with this immunity. Although it is unanimous that vaccination (whatever the scheme) helps with immunization, there are reports of different efficacy [43]. Accordingly, we observed distinct values of anti-IgG depending on the administrated vaccine.

Patients with an autoimmune disease presented lower levels of anti-S IgG (*p* = 0.030) and smokers presented lower values than never-smokers (*p* = 0.024). Vaccination against SARS-COV-2 in patients with a previous immune disease was the result of much analysis, and it was always considered a risk [44]. A study carried out with autoimmune patients (multiple sclerosis), showed that the levels of antibodies [45] are similar to those described in the present work. A similar situation occurs with smokers, presenting a reduction in the levels of antibodies [46]. The severity of COVID-19 did not affect the concentration of anti-S IgG.

Given that this work was performed through distinct phases of the COVID-19 pandemic, our results may have been influenced by different strains of SARS-CoV-2, vaccine availability and adhesion, antiviral treatment administrations’ evidence and accessibility, as well as the literacy of the population regarding this infectious disease, which varied throughout the years. A potential limitation of our study is also the inability to verify the existence of concomitant viral pathologies.

The aim of this retrospective study was to describe the health status and to characterize the severity of the disease caused by SARS-CoV-2 infection in people living in an inland Portuguese area. The results obtained in this study open new perspectives for future work unraveling the pre- and post-COVID health status of this population.

## 5. Conclusions

To sum up, this study focused on an inland region of Portugal. The health statuses of the population and the epidemiological data about COVID-19 were described and characterized. Associations were found between specific traits and some pathologies. The main highlight is the enormous adherence to vaccination, as demonstrated by more than 97% of the individuals being vaccinated, thus showing that the Portuguese population (even in the considered region) realized the importance of vaccination and had the means at their disposal to obtain this vaccination [39].

## Figures and Tables

**Table 1:** Characteristics of the population under study.

Variable		Frequency ^a^	Mean ^b^
Age (years)			48.95 [48.23–49.67]
Sex	Male	64.0% (994)	
	Female	36.0% (558)	
Weight (kg)			72.41 [71.66–73.18]
Height (m)			1.66 [1.65–166]
BMI (m/kg^2^)			26.28 [26.04–26.51]
BMI Category	Underweight	1.5% (24)	
	Normal	41.3% (642)	
	Overweight	37.4% (581)	
	Class 1 Obesity	15.0% (233)	
	Class 2 Obesity	3.2% (50)	
	Severe obesity (Class 3)	1.5% (23)	
Systolic Arterial Blood Pressure (mmHg)			126.01 [125.17–126.85]
Diastolic Arterial Blood Pressure (mmHg)			80.23 [79.73–80.73]
Peripheral oxygen saturation (%)			97.75 [97.71–97.80]
Blood Type (N = 631)	0 +	31.7% (200)	
	0 −	8.4% (53)	
	A +	41.5% (262)	
	A −	7.8% (49)	
	B +	5.2% (33)	
	B −	2.2% (14)	
	AB +	2.7% (17)	
	AB −	0.5% (3)	
Smoking status (N = 1501)	Current smoker	18.9% (284)	
	Former smoker	24.5% (368)	
	Never smoker	56.6% (849)	
**SARS-CoV-2 Infection ^c^**	**Yes**	**32.6% (505)**	
	**No**	**67.4% (1046)**	

^a^ For categorical variables, values are expressed as % (n). ^b^ For continuous variables, values are expressed as mean [95% Confidence Interval]. ^c^ These results are highlighted in bold according to the goals of this study.

**Table 2:** Prevalence of diseases in the studied population.

Area	Disease ^a^	Absolute Frequency (n)	Relative Frequency (%)
Cardiovascular diseases	Arterial hypertension (N = 1553)	298	19.2%
	Heart failure (N = 1522)	15	1.0%
	Arrhythmia (N = 1551)	26	1.7%
	Ischemic disorder (N = 1552)	18	1.2%
	Coronary artery disease (N = 1521)	28	1.8%
	Cerebrovascular disease (N = 1553)	9	0.6%
Nephrology	Chronic Kidney Disease (N = 1523)	16	1.0%
Endocrinology	Dyslipidemia (N = 1553)	196	12.6%
	Diabetes Mellitus (N = 1553)	102	6.6%
	Thyroid disorder (N = 1552)	79	5.1%
Pulmonology	Pulmonary disease (N = 1552)	104	6.7%
	Asthma (N = 1553)	56	3.6%
	Obstructive sleep apnea syndrome (N = 1553)	15	1.%
Oncology	Oncological disease (N = 1547)	26	1.7%
Hematology	Hematological disease (N = 1553)	17	1.1%
Psychiatry	Psychiatric disease (N = 1553)	9	0.6%
Immuno-Allergology	Autoimmune disease (N = 1538)	72	4.7%
	Allergies (N = 1493)	45	3.0%

^a^ Total number of subjects in each disease varied due to missing data.

**Table 3:** Patients’ receiving treatment for the most frequent comorbidities: arterial hypertension and dyslipidemia.

Disease	Total	Under Treatment (n)	Under Treatment (%)	*p*-Value
Arterial Hypertension	19.2% (298)	264	88.6% (264/298)	**0.052 ^a^**
Dyslipidemia	12.6% (196)	106	54.1% (106/196)	

^a^ Significant results highlighted in bold.

**Table 4:** Distribution of COVID-19 categories of severity.

Severity of the Infection	Absolute Frequency (n)	Relative Frequency (%)	Admitted to the ICU
Asymptomatic	73	14.5%	0
Mild	354	70.1%	0
Moderate	71	14.1%	0
Severe	7	1.4%	2

**Table 5:** Comparison of the severity of first-time and second-time infections of SARS-CoV-2.

	Severity of First Time Infected ^a^	Severity of Second Time Infected ^a^	*p*-Value
Asymptomatic	5 (31.25%)	5 (31.25%)	**0.038 ^b^**
Mild	8 (50.0%)	9 (56.25%)	
Moderate	2 (12.5%)	2 (12.5%)	
Severe	1 (6.25%)	0 (0%)	
Total	16 (100%)		

^a^ Values are expressed as n (%).^b^ Significant results highlighted in bold.

**Table 6:** Vaccines administrated against SARS-CoV-2 infection and relative immunization (anti-Spike protein IgG concentration in the sera of the individuals).

			Comparison of Antibody Concentration (*p*-Value)									
Vaccine	Frequency n (%)	Antibody Concentration (AU/mL) *	Pfizer	Moderna	AstraZeneca	Janssen	Pfizer/Moderna	Pfizer/AstraZeneca	Pfizer/Janssen	Moderna/AstraZeneca	Moderna/Janssen	CoronaVac
Pfizer ^a^	964 (64.3)	377.29 [365.11–389.48]	-	NS ***	*p* < 0.001 ****	***p* < 0.001**	NS	***p* = 0.006**	NS	***p* = 0.004**	NS	NS
Moderna ^b^	171 (11.4)	424.92 [403.08–446.75]	-	-	***p* < 0.001**	***p* < 0.001**	NS	NS	NS	NS	NS	NS
AstraZeneca ^c^	100 (6.7)	161.82 [118.25–205.38]	-	-	-	NS	***p* < 0.001**	***p* < 0.001**	***p* < 0.001**	***p* < 0.001**	***p* < 0.001**	NS
Janssen ^d^	41 (2.7)	233.30 [157.73–308.88]	-	-	-	-	***p* = 0.018**	***p* < 0.001**	***p* < 0.001**	***p* < 0.001**	***p* < 0.001**	NS
Pfizer/Moderna	12 (0.8)	462.29 [404.30–520.29]	-	-	-	-	-	NS	NS	NS	NS	NS
Pfizer/AstraZeneca	103 (6.9)	455.19 [432.10–478.28]	-	-	-	-	-	-	NS	NS	NS	NS
Pfizer/Janssen	24 (1.60)	462.01 [410.32–513.71]	-	-	-	-	-	-	-	NS	NS	NS
Moderna/AstraZeneca	61 (4.10)	480.88 [458.18–503.58]	-	-	-	-	-	-	-	-	NS	NS
Moderna/Janssen	22 (1.50)	463.42 [412.68–514.16]	-	-	-	-	-	-	-	-	-	NS
CoronaVac ^e^	1 (0.10)	470.50 **	-	-	-	-	-	-	-	-	-	-

^a^ BTN162b2—Pfizer/BioNTech. ^b^ mRNA-1273—Moderna. ^c^ AZD1222—Oxford/AstraZeneca. ^d^ Ad26.COV2.S—Janssen (Johnson & Johnson). ^e^ CoronaVac—Sinovac. * Values are expressed as mean [95% Confidence Interval]. ** Confidence interval not presented as only one participant was inoculated with this vaccine. *** NS–statistically non-significant. **** Results with statistical significance are highlighted in bold.

**Table 7:** Immunization (anti-Spike protein IgG concentration in the sera of the individuals) according to the vaccine technology.

Vaccine Technology	Antibody Concentration (AU/mL) ^a^	Vs. Other Technology	Adjusted *p*-Value ^e^
Viral vector vaccines	182.75 [144.94–220.56]	Whole-virus vaccines	1.000
		Gene vaccines	**<0.001**
		Mix ^c,d^	**<0.001**
Whole-virus vaccines	470.50 ^b^	Gene vaccines	1.000
		Mix ^c,d^	1.000
Gene vaccines	385.21 [374.40–396.01]	Mix ^c,d^	**<0.001**

^a^ Values are expressed as mean [95% Confidence Interval]. ^b^ Values are expressed as mean [95% Confidence Interval]. ^c^ Mix, Combinations/mixture of vaccines based on different technologies. ^d^ Antibody concentration (AU/mL) = 464.47 [449.54–479.40]. ^e^ Significant results highlighted in bold.

## Data Availability

No new data were created or analyzed in this study. Data sharing is not applicable to this article.
